# The Effects of Dietary n-3 Highly Unsaturated Fatty Acids on Growth, Antioxidant Capacity, Immunity, and Oxylipin Profiles in *Acipenser dabryanus*

**DOI:** 10.3390/antiox13040421

**Published:** 2024-03-29

**Authors:** Jinping Wu, Wei Xiong, Wei Liu, Jinming Wu, Rui Ruan, Peng Fu, Yuqi Wang, Yuan Liu, Xiaoqian Leng, Pengcheng Li, Jia Zhong, Chuang Zhang, Hao Du

**Affiliations:** 1 Yangtze River Fisheries Research Institute, Chinese Academy of Fishery Sciences, Wuhan 430223, China; wujinping@yfi.ac.cn (J.W.); taihuxiongwei@163.com (W.X.); liuwei@yfi.ac.cn (W.L.); jinming@yfi.ac.cn (J.W.); ruanrui@yfi.ac.cn (R.R.); w577295817@163.com (Y.W.); lyuan@yfi.ac.cn (Y.L.); lengxiaoqian@yfi.ac.cn (X.L.); pcli@yfi.ac.cn (P.L.); zhongjia@yfi.ac.cn (J.Z.); 2 Chongqing Fishery Sciences Research Institute, Chongqing 400020, China; fupeng592013@163.com

**Keywords:** *Acipenser dabryanus*, dietary n-3 HUFA levels, growth performance, antioxidant, immunity capacity, serum oxylipin

## Abstract

Currently, the effects of dietary levels of n-3 highly unsaturated fatty acids (HUFAs) on the growth performance, antioxidant capacity, immunity, and serum oxylipin profiles of female F_2_-generation Yangtze sturgeon remain unknown. A total of 75 Yangtze sturgeons, an endangered freshwater fish species, with an average body weight of 3.60 ± 0.83 kg, were randomly allocated to 15 concrete pools, with each dietary group represented by 5 fish per pool. The fish were fed five different experimental diets containing various levels of n-3 HUFAs (0.5%, 1.0%, 1.5%, 2.0%, and 2.4%). After a feeding period of 5 months, no significant differences in the growth performances of the fish were observed among the five dietary groups (*p* > 0.05). However, we did note that the serum levels of low-density lipoprotein cholesterol (LDL-C), triglycerides (TGs), and total cholesterol (TCHO) exhibited a marked increase in the fish that consumed higher dietary n-3 HUFA levels (*p* < 0.05). Conversely, alkaline phosphatase (ALP) activities showed a notable decrease as dietary n-3 HUFA levels increased (*p* < 0.05). Serum antioxidant indices, such as the activity levels of superoxide dismutase (SOD) and glutathione peroxidase (GSH-Px), were significantly higher in the 2.4% HUFA group compared to the 0.5% HUFA group. Additionally, muscle antioxidant indices, including total antioxidant capacity (T-AOC), catalase (CAT), and SOD activity, exhibited notable increases as dietary n-3 HUFA levels increased (*p* < 0.05). Furthermore, there was a decrease in malondialdehyde (MDA) levels as dietary n-3 HUFA levels increased (*p* < 0.05). In relation to immune indices, only serum immunoglobulin M (IgM) and muscle complement 3 (C3) were found to be influenced by dietary n-3 HUFA levels (*p* < 0.05). A total of 80 oxylipins were quantified, and our subsequent K-means cluster analysis resulted in the classification of 62 oxylipins into 10 subclasses. Among the different n-3 HUFA diets, a total of 14 differential oxylipins were identified in the sera. These findings demonstrate that dietary supplementation with n-3 HUFAs exceeding a 1.0% level can enhance antioxidant capacity and regulate serum lipid metabolism, potentially through modulation of oxylipins derived from ARA, DHA, and EPA. These insights provide novel perspectives on the mechanisms underlying these observations.

## 1. Introduction

Omega-6 arachidonic acid (ARA, C20:4n6), omega-3 eicosapentaenoic acid (EPA, 20:5n-3), and docosahexaenoic acid (DHA, 22:6n-3) are essential long-chain highly unsaturated fatty acids (LC-HUFAs) that play pivotal roles in the regulation of growth performance, antioxidant capacity, lipid metabolism, and reproduction [1,2,3,4]. It is widely acknowledged that freshwater fish and salmon possess the ability to biosynthesize biologically active LC-PUFAs, such as n-3 EPA, DHA, and n-6 ARA, within their bodies [5]. However, they exhibit a limited or no capacity for conversion of C18 PUFA into LC-PUFAs due to potential constraints in desaturation and/or elongation enzymes [6,7]. Consequently, the inclusion of these fatty acids in the diet of fish has become particularly indispensable. Bell et al. [8] presented evidence indicating a diminished capacity for synthesizing LC-PUFAs in aging fish; a similar tendency has been observed in humans. Tocher et al. [9] also reported that freshwater fish may not meet their essential fatty acid requirements within their diet, potentially leading to reproductive failure. In the case of sturgeons, the white sturgeon (*Acipenser transmontanus*) and the Russian sturgeon (*A. gueldenstaedtii*) require n-3 and n-6 fatty acids [10,11]. Furthermore, Luo et al. [12] observed improved fecundity, egg hatchability, and overall larval quality in *A. baeri* female broodstock fed with high levels of DHA. Subsequently, Luo et al. [13] emphasized the significance of enhancing DHA/EPA ratios in larvae, as they selectively accumulate more DHA, followed by EPA and ARA, rather than MUFAs, to meet early developmental requirements. Recently, Yoon et al. [14] have reported the crucial role of EPA and DHA in the early life stages of lake sturgeon (*A. fulvescens*) with regard to their growth and survival. It is imperative that they receive appropriate quantities of LC-PUFAs through their diet, particularly during specific periods of sturgeon growth, because of their indispensable structural and functional significance within these organisms’ physiology.

*Acipenser dabryanus*, commonly referred to as the Yangtze sturgeon, is an indigenous species in China and is primarily found in the mainstem of the upper Yangtze River and its tributaries. In recent decades, because of the construction of cascade dams, overfishing, and habitat degradation, there has been a significant decline in the population of Dabry’s sturgeon [15,16]. Regrettably, this decline has led to a cessation of their natural reproductive behavior by 2000, and it became evident that the natural population could no longer be sustained [17]. Consequently, this sturgeon species was classified as critically endangered on the International Union for Conservation of Nature (IUCN) Red List [18]. Numerous efforts have been made to rescue this species [19,20,21,22,23,24,25]; however, progress remains slow. The gonad stage II plays a crucial role in the developmental process of Yangtze sturgeons, which often remain in this stage for an extended period of time. Presently, nutritional research on this species is extremely limited. Furthermore, the available data regarding dietary n-3 HUFAs in the F_2_ generation of Yangtze sturgeon are insufficient. Only Qiao et al. [26] have reported on the distribution of nutrients in different tissues of subadult Dabry’s sturgeon; they indicated that EPA and DHA may play crucial roles in gonad development. Additionally, DHA and EPA exhibit significant physiological effects, which are often mediated by their oxylipins. However, there is a scarcity of metabolism studies examining the combined impact of DHA and EPA supplementation on oxylipin patterns in fish species. In other terrestrial animals, it has been reported that gender can affect oxylipin status. Consequently, female Yangtze sturgeon were the experimental subjects in the current study to provide more precise information for future lipid research. Analyzing oxylipins derived from specific fatty acids can provide valuable insights into the biological effects of dietary lipids beyond their influence on fatty acid tissue profiles. We hypothesized that incorporating dietary n-3 highly unsaturated fatty acids at an appropriate dosage could enhance antioxidant capacity and influence the oxylipin profile. Therefore, the objective of this trial was to assess the regulatory effects of dietary n-3 highly unsaturated fatty acids on the growth performance, antioxidant capacity, and oxylipin status in the F_2_ generation of Yangtze sturgeon.

## 2. Materials and Methods

### 2.1. Experimental Diet

The subjects’ diet formulation, proximate composition, and fatty acid profiles are presented in Table 1 and Table 2. Five isonitrogenous and isoenergetic diets were formulated to contain approximately 42.54% crude protein and 10.68% crude lipid. Fish meal, soybean meal, chicken meal, and squid meal were used as the main protein sources. 

Wheat flour was used as the carbohydrate source. The lipid base comprised soybean oil, arachidonic acid oil (ARA), and eicosapentaenoic acid (EPA) and docosahexaenoic acid (DHA) oils in different amounts, with a gradual increase in DHA and EPA being prioritized in the diet formulations; final proportions of 6.18% (0.50%), 11.45 (1.00%), 15.53% (1.50%), 22.17% (2.00%), and 26.9 (2.40%) of total fatty acids were used. The EPA and DHA oils were derived from fish oil. The EPA oil content was 78.44%, and the main fatty acid profile of the EPA oil contained eicosanoic acid at 19.91%. The DHA oil content was 98.23%. The DHA and EPA oils were purchased from Shaanxi Pioneer Biotech Co., Ltd., Shanxi, Hanzhong, China. The ARA oil was purchased from Fuxing Biotechnology Co., Ltd., Hubei, Hanchuan, China. The raw materials of fish meal, soybean meal, chicken meal, squid meal, and wheat flour were purchased from Jinjia feed Co., Ltd., Zhejiang, Hangzhou, China. The diets were pelleted using a medium-sized laboratory meat grinder (MM32, Hunan Hengji Machinery Co., Ltd., Hunan, Yiyang, China); the pellets were 8 mm in diameter and approximately 20.0 mm in length, and they were stored at −20 °C until being fed to the Yangtze sturgeon.

### 2.2. Fish and Feeding Trial 

At the Taihu Experimental Station of the Yangtze River Fisheries Research Institute in Jingzhou, Hubei province, 75 female sturgeon were randomly selected and cultured in concrete pools (length: 83.5 m; width: 2.0 m; and depth: 2.0 m) with a continuous groundwater and lake water complex flow system and continuous aeration. Prior to the experiment, these sturgeon had stage II gonads and an average body weight of 3.60 ± 0.83 kg. The selection of stage II experimental fish was primarily based on age, and three fish were randomly selected from a batch of experimental fish for HE staining to ensure the accuracy of our assessment of ovary development. Ovaries were fixed in 4% paraformaldehyde solution overnight, and then transferred to 70% ethanol until ready for paraffin sectioning. Slices with a thickness of 5 μm were obtained using a standard paraffin-embedding method, then stained with hematoxylin–eosin (HE). Images of the sections were captured using a Nikon Eclipse E100 (Tokyo, Japan) microscope equipped with a Nikon DS-U3 (Tokyo, Japan) imaging system. Histological sections of the ovaries are shown in Supplementary Figure S1. Yangtze sturgeon were randomly distributed into 15 indoor concrete pools (diameter: 3 m; depth: 0.5 m; n = 5/tank). A pair of female-specific primers (F: TAATCAATTGTAAGTCGCCAAG; R: ATTTTATTACGGTGAGTATACGAA) from our laboratory were used for sex identification, following the detailed process described in an existing report [27]. Bright bands represent females, while no bands represent males; an amplified band is exhibited in Supplementary Figure S2. Throughout the experiment, the flow rate of the water was approximately 10.2 L/min. The water temperature varied between 17.4 °C and 22.2 °C (mean: 20.10 °C). The dissolved oxygen level of the water varied between 5.71 mg/L and 9.09 mg/L (mean: 7.42 mg/L). The pH of the water varied between 7.98 and 8.12 (mean: 8.04). The NH_4_-N concentration of the water varied between 0.07 mg/L and 0.28 mg/L (mean: 0.15 mg/L). Experimental fish were hand-fed 8 mm pellets to satiation twice daily (8:00 and 20:00) for five months. The feeding rate of the Yangtze sturgeon was approximately 0.8% of their body weight and adjusted on the basis of the weather conditions and the status of fish feeding. 

### 2.3. Sample Collection and Analysis 

#### 2.3.1. Growth Performance, Biochemical, and Immunity Index Analysis

After the feeding trial was completed, we did not feed the fish for 24 h. All surviving Yangtze sturgeon were collected from each concrete pool, counted, measured, and weighed. Two sampled fish from each tank were anaesthetized with MS-222 (GREENHX Biological Technology Co., Ltd., Beijing, China). After their body length was measured, 2 mL of blood was collected using a 5 mL syringe. The blood was then allowed to clot overnight in a 4 °C refrigerator. Serum samples were collected via centrifugation (3000× *g*/min 4 °C, 10 min), and they were placed in a −80 °C freezer to proceed with the biochemical indices, antioxidant capacity, immunity, and oxylipin analyses. 

Back muscle samples were obtained using a copper probe (diameter: 5 mm; length: 16 cm) with a long and notched tube (diameter: 4 mm; length: 20 mm) on the end, and samples were placed in the −80 °C freezer for antioxidant capacity and immunity analyses. 

To reduce the large differences in biological replicates, the serum and muscle samples used in the analyses of their biochemical indices, antioxidant capacity, immunity, and oxylipin were mixed samples taken from two fish; therefore, each treatment had three biological replicates.

Growth-related indices were estimated using the following formulas:

Survival rate (SR, %) = (final fish number/initial fish number) × 100;

Weight gain rate (WGR, %) = (final weight − initial weight)/initial weight × 100;

Feed conversion rate (FCR) = dry feed intake/wet weight gain;

Condition factor (CF, %) = 100 × body weight (g)/body length^3^ (cm).

The levels of total antioxidant capacity (T-AOC), total superoxide dismutase (T-SOD), glutathione peroxidase (GSH-Px), and catalase (CAT) in the serum and muscle, as well as the levels of lipid peroxidation metabolite malondialdehyde (MDA), complement 3 (C3), complement 4 (C4), and immunoglobulin M (IgM), were determined using commercial kits, following the manufacturer’s instructions (Nanjing Jiancheng Bioengineering Institute, Nanjing, China).

The activity levels of serum aspartate aminotransferase (AST), alanine aminotransferase activity (ALT), alkaline phosphatase (ALP), serum total protein (TP), albumin (ALB), triglycerides (TGs), total cholesterol (TC), high-density lipoprotein cholesterol (HDL-C), low-density lipoprotein cholesterol (LDL-C), and glucose (GLU) were measured using an automatic biochemical analyzer (BS-460; Shenzhen Mindray Biomedical Electronics Co., Ltd. Guangdong, Shenzhen, China).

The dietary crude protein, crude lipid, and ash contents were detected using the methods of AOAC (1995) [28]. The dietary moisture content was analyzed by oven drying at 105 °C. The detection of dietary fatty acids was carried out following the methods of Wu et al. [29].

#### 2.3.2. Serum Oxylipin Analysis

With respect to the HPLC conditions, the sample, extracts were analyzed using an LC-ESI-MS/MS system (ultraperformance liquid chromatography, UPLC; ExionLC^TM^ AD; MS, QTRAP^®^ 6500+ System, Boston, MA, USA). The analytical conditions were as follows: HPLC—column, Waters ACQUITY UPLC HSS T3 C18 (100 mm × 2.1 mm i.d, 1.8 µm); solvent system—water with 0.04% acetic acid (A) and acetonitrile with 0.04% acetic acid (B). The gradient was 0–2.0 min from 0.1% to 30% B; 2.0–4.0 min at 50% B; and 4.0–5.5 min at 99% B, which was maintained for 1.5 min and at 6.0–7.0 min. It then reduced to 0.1% B and was maintained for 3.0 min. The flow rate was 0.4 mL/min; the temperature was 40 °C; and the injection volume was 10 μL. Regarding the ESI-MS/MS conditions, the ESI sources’ operating parameters were as follows: ion source, ESI; source temperature, 550 °C; and ion spray voltage (IS), −4500 V. The curtain gas (CUR) was set at 35 psi.

### 2.4. Statistical Analysis

The data in the present study are shown as the means ± SE (standard error) and were subjected to a one-way ANOVA after data normality and homogeneity were verified using IBM 22.0 (IBM Corp., Michigan Avenue, Chicago, IL, USA). If the data did not exhibit homogeneity of variance, a nonparametric test (Kruskal–Wallis) was used for the comparison. The differences among treatments were identified, and Tukey’s honest significant difference (HSD) test was used to compare the mean values; values of *p* < 0.05 were considered significant.

## 3. Results

### 3.1. Growth Performance and Morphological Parameters

The different dietary n-3 HUFA levels did not affect the FBW, FBL, WGR, FCR, CF, and survival rate (SR) (*p* > 0.05) (Table 3).

### 3.2. Serum Biochemical Indices

The serum biochemical indices are shown in Table 4. The different dietary n-3 HUFA levels did not affect serum AST, ALT, ALB, TP, GLU, and HDLC (*p* > 0.05). The serum activities of ALP reached their highest levels in fish fed with the 0.5% n-3 HUFA diet, and they were significantly higher than in fish fed with the 2.0% and 2.4% n-3 HUFA diets (*p* < 0.05). The contents of TCHO and LDLC in the serum increased, along with an increase in n-3 HUFA levels, and the contents of TCHO and LDLC in fish fed with the 1.5%, 2.0%, and 2.4% n-3 HUFA diets were significantly higher than in the fish fed with the 0.5% n-3 HUFA diet (*p* < 0.05). The TG contents reached their highest levels in the fish fed with the 1.5% n-3 HUFA diet, and they were significantly higher than in the other groups (*p* < 0.05).

### 3.3. Serum Antioxidant and Immunity Status

The activity of enzymes in the serum of Yangtze sturgeon is shown in Table 5. The T-AOC and CAT activities and MDA contents were not affected by the different contents of dietary n-3 HUFAs (*p* > 0.05). With an increase in the n-3 HUFA levels in the diet, the activity of SOD increased, reaching the maximum value in the fish fed the 2.4% n-3 HUFA diet; it was significantly higher than in the fish fed the 0.5% n-3 HUFA diet (*p* < 0.05). The GSH-Px activities in the fish fed the 2.4% n-3 HUFA diet were significantly higher than in the fish fed the 0.5% and 1.5% n-3 HUFA diets (*p* < 0.05).

With an increase in the n-3 HUFA levels in the diet, the contents of IgM increased, reaching the maximum value in the fish fed the 1.0% n-3 HUFA diet; they were also significantly higher than in the fish fed the 0.5% n-3 HUFA diet (*p* < 0.05).The serum contents of C3 and C4 were not affected by dietary n-3 HUFAs (*p* > 0.05).

### 3.4. Muscle Antioxidant and Immunity Status

The activity of enzymes in the muscles of Yangtze sturgeon is shown in Table 6. With an increase in the n-3 HUFA levels in the diet, the activity of T-AOC increased, reaching a maximum value in fish fed the 2.0% n-3 HUFA diet; it was significantly higher than in the 0.5%, 1.0%, and 1.5% n-3 HUFA diet groups (*p* < 0.05). The activity of SOD also increased, reaching a maximum value in the 1.0% n-3 HUFA diet group; it was significantly higher than in the 0.50% and 2.4% n-3 HUFA diet groups (*p* < 0.05). The CAT activities in the 1.0% n-3 HUFA diet group were significantly higher than in the fish fed the 0.5% and 2.4% n-3 HUFA diets (*p* < 0.05). The content of MDA first increased and then decreased, reaching the minimum value in the 2.4% n-3 HUFA diet group; it was significantly lower than in the 0.5%, 1.0%, and 1.5% n-3 HUFA diet groups (*p* < 0.05).

The muscle contents of IgM and C4 were not affected by the dietary n-3 HUFAs (*p* > 0.05). With an increase in the n-3 HUFA levels in the diet, the content of C3 decreased, reaching the minimum value in the fish fed the 2.4% n-3 HUFA diet; it was significantly lower than in the fish fed the 1.0% and 1.5% n-3 HUFA diets (*p* < 0.05).

### 3.5. Serum Oxylipin Profiles

The total ion current (TIC) overlap plot of the quality control samples (Supplementary Figure S3A), an integral correction diagram (Supplementary Figure S3B), and a canonical plot (Supplementary Figure S3C) are provided to ensure the accuracy of all oxylipins during the period of analysis. In the current study, a total of 80 oxylipins were detected in the serum of the Yangtze sturgeon (Supplementary Table S1).

These 80 oxylipins are mainly classified into seven categories; most were derived from ARA (38.75%), and the second most oxylipins were derived from DHA (20.0%). EPA-derived oxylipins ranked third (15.0%), followed by linoleic acid (LA) (11.25%), dihomo-γ-linolenic acid (DGLA) (5.00%), α-linolenic acid (ALA) (5.00%), and γ-linolenic acid (GLA) (1.25%). Detailed information about each group can be found in Supplementary Table S1.

**Figure 1:**
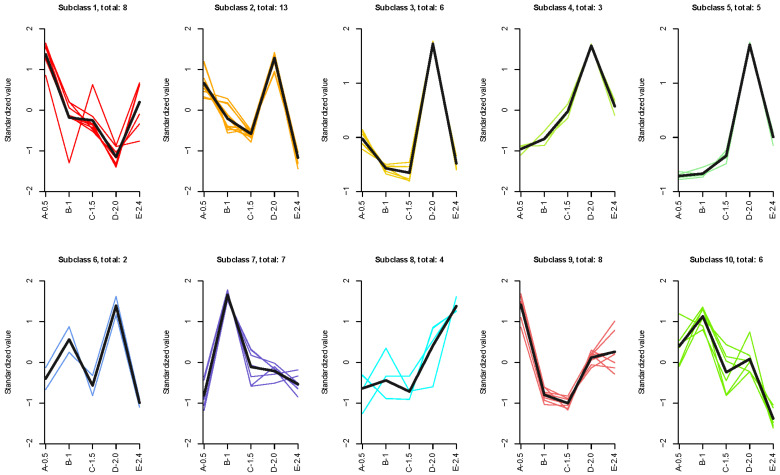
K-means clustering of serum oxylipin in female F_2_-generation Yangtze sturgeon fed diets containing different levels of n-3 HUFAs.

After K-means cluster analyses, 62 oxylipins were clustered into 10 subclasses (Figure 1). Detailed classifications of these 62 oxylipins are presented in Supplementary Table S2.

The 80 oxylipins were analyzed using IBM 22.0 software (IBM Corp., Michigan Avenue, Chicago, IL, USA), and 14 different oxylipins were identified across the various n-3 HUFA diet groups (Table 7). The serum contents of PGA2 in the 2.0% n-3 HUFA diet were significantly lower than those in the 0.5% n-3 HUFA diet (*p* < 0.05). The contents of 18-HETE and 17-HETE in the 2.0% n-3 HUFA diet group were significantly higher than those in the 0.5% n-3 HUFA diet group (*p* < 0.05). The contents of D-γ-LA in the 2.4% n-3 HUFA diet group were significantly lower than those in the 1.0% n-3 HUFA diet group (*p* < 0.05). The contents of 16-HDHA, 10-HDHA, 14(S)-HDHA, 7-HDHA, 8-HDHA, and 4-HDHA in the 1.0% n-3 HUFA diet group were significantly higher than those in the 0.5% n-3 HUFA diet group (*p* < 0.05). The contents of 5-HEPE and 14(15)-EpETE in the 2.0% n-3 HUFA diet group were significantly higher than those in the 0.5% n-3 HUFA diet group (*p* < 0.05). The contents of EPA in the 2.4% n-3 HUFA diet group were significantly higher than those in the 0.5% n-3 HUFA diet group (*p* < 0.05). The contents of γ-LA in the 2.4% n-3 HUFA diet group were significantly lower than those in the 1.0% n-3 HUFA diet group (*p* < 0.05).

## 4. Discussion

In this study, a feeding trial was conducted for up to 5 months to investigate the effects of n-3 HUFAs on Yangtze sturgeon. Interestingly, we found that this supplementation did not impact on important parameters such as WGR, FCR, CF, and SR. However, it is important to note that our conclusion remains unchanged—there is still a need to include EPA and DHA in the diets of sturgeon. This conclusion aligns with the findings of Luo et al. [12], who demonstrated that female Siberian sturgeon broodstocks with high levels of DHA experienced improved fecundity, egg hatchability, and, overall, enhanced larval quality. The authors also emphasized the significance of optimizing DHA/EPA ratios for optimal early development in Siberian sturgeon [13]. Yoon et al. [14] reported that EPA and DHA play crucial roles in the growth and survival of lake sturgeon during their first year. Overall, we concluded that n-3 HUFAs have a significant role to play in broodstocks and in the early stages of sturgeon growth. Different developmental stages exhibit variations in nutrient utilization and selection. Asil et al. [30] pointed out that the rates of growth in the larval and juvenile stages are higher than in the maturation stage, wherein energy expenditure shifts away from weight gain and toward reproduction. In our study, the experimental fish were in ovarian stage II, which represents a significant developmental period relative to the lifespan of sturgeons. Although growth performance parameters were not affected by n-3 HUFAs, this does not mean that other physiological functions are not regulated by n-3 HUFAs. Certainly, the current feeding period of 5 months is relatively short for Yangtze sturgeons, which have an average initial body weight of 3.60 ± 0.83 kg. Additionally, it should be noted that the control group in this study contained 6.18% n-3 HUFAs, which may potentially mask the impact that a deficiency in n-3 HUFAs can have on female Yangtze sturgeons. Future studies should explore the effects of a diet lacking n-3 HUFAs and extended feeding periods on female Yangtze sturgeon to further elucidate their nutritional requirements. Similar observations have been made in juvenile Atlantic salmon (*Salmo salar*) [31], juvenile golden pompano (*Trachinotus ovatus*) [32], and turbot (*Scophtalmus maximus* L.) [33].

Serum biochemical indices serve as crucial indicators that reflect the health status of fish. A significant decrease in serum glucose (GLU) and total protein (TP) levels, along with elevated activities of alanine transaminase (ALT) and aspartate transaminase (AST), was reported by Lee [34] in rockfish (*Sebastes schlegeli*); these results were a consequence of dietary n-3 LC-PUFA deficiency. Furthermore, in starry flounder (*Platichthys stellatus*), an increase in AST activities was observed, whereas no impact was noted on serum ALT activities or GLU and TP concentrations, as reported by Lee [35]. Mozanzadeh et al. [36] found relatively lower levels of serum GLU in fish fed diets containing 0.1% and 0.6% n-3 LC-PUFAs when comparing them with other groups; however, these diets did not affect serum TP and albumin (ALB) concentrations. However, ALP exhibited a contrasting pattern. Under the current experimental conditions, the serum levels of GLU, TP, and ALB concentrations and the activities of ALT and AST were consistent across all groups. However, a decrease in ALP activities accompanied the increase in dietary n-3 HUFA levels. These findings not only shed light on variations among different fish species but also indicate that the current diet for this species containing 6.18% n-3 HUFAs is sufficient and does not lead to any relevant deficiencies. Interestingly, the fish fed diets with 6.18% n-3 HUFAs had lower serum triglycerides (TGs) and LDL-C levels compared to the other groups; this observation contradicts the findings of previous studies conducted on turbot [33], silvery-black porgy (*Sparidentex hasta*) [36], juvenile Pacific white shrimp (*Litopenaeus vannamei*) [37], and Atlantic salmon [38]. Simultaneously, lower serum TCHO contents were found in fish fed diets with 6.18% n-3 HUFAs. Similar phenomena were also observed in juvenile black seabream (*Acanthopagrus schlegelii*) and starry flounder [35,39]. Lower levels of serum TCHO in fish fed diets with 6.18% n-3 HUFAs might be a consequence of the increased soybean oil content involved in the preparation of this diet. This is because, as Castro et al. [40] observed, cholesterol concentrations are lower in fish fed with vegetable oil (that has lower dietary n-3 HUFA levels) than fish fed with fish oil (that has higher dietary n-3 HUFA levels). One possible explanation for this difference is that the vegetable oil diets contain less cholesterol than fish oil; the levels observed in the control group can also be attributed to these oils themselves, as they are a major factor influencing cholesterol levels in fish fed such diets [39].

The antioxidant capacity of fish is primarily reflected through various antioxidant enzymes, which protect the fish body from damage by eliminating excessive reactive oxygen species (ROS) [41]. Oxidative stress, a state of imbalance between oxidation and antioxidation, can have adverse effects on the fish’s health [42]. MDA serves as the primary product of lipid peroxidation [43], while the content of T-AOC represents the overall antioxidant level within the fish’s body. Enzymes such as SOD, CAT, and GPx play a pivotal role in scavenging free radicals and alleviating oxidative stress [44]. Under the experimental conditions of this study, it was observed that when the dietary content of n-3 HUFAs reached 2.4%, the activity of SOD in serum peaked. However, in muscle, this peak activity was achieved with a diet containing 1.0% n-3 HUFAs. This suggests that the optimal dietary requirements for n-3 HUFAs vary depending on the specific antioxidant enzyme and tissue type. Similarly, the significant increases in the CAT and TAC activities in serum required higher concentrations of n-3 HUFAs, whereas in muscle, a significant enhancement in these enzyme activities was observed in the fish fed diets containing 1% and 2% n-3 HUFAs compared to the control group. Notably, the muscle MDA content was significantly lower in fish fed diets with 2% to 2.4% n-3 HUFAs compared to the control group. In summary, the dietary requirements for n-3 HUFAs to exert different antioxidant enzyme activities in various tissues range from 1% to 2%. When the dietary n-3 HUFA content reaches 2%, the fish demonstrated a remarkable ability to eliminate lipid peroxides and maintain their overall health. Significantly increased T-AOC activities in the 1.24% group were observed in golden pompano fed diets with different n-3 HUFA levels [32]. In the observation of largemouth bass (*Micropterus salmoides*) [3], slightly larger values of MDA were observed in fish fed diets with 1% compared to 2% EPA + DHA. Meanwhile, the serum contents of IgM and muscle C3 demonstrated an increase with 1%-1.5% n-3 HUFAs, followed by a decreasing trend at 2.4% n-3 HUFAs. Similar to the observations of juvenile golden pompano [32], they noted that the levels of IL-1β and IL-6 in serum and the gene mRNA expression of *nf-κb*, *il-1β*, *il-6*, and *il-8* in the liver all decreased with an increase in the n-3 HUFA levels up to 2.1%. These results indicate that dietary supplementation with appropriate n-3 HUFA contents can effectively enhance the immune ability of fish.

Many studies have reported on oxylipin profiles in various species, including humans, rats, and even nonmammals. To the best of our knowledge, the effects of dietary long-chain PUFAs on fish oxylipin profiles have not been studied yet. In our study, we detected 80 oxylipins after feeding n-3 HUFA diets to Yangtze sturgeon for up to 5 months. Interestingly, 38.75% of these oxylipins were derived from ARA, indicating the majority. These findings are consistent with those observed in yellow croaker and rainbow trout [45]. Additionally, a study conducted on rat hearts identified 75 oxylipins, two-thirds of which were derived from n-6 PUFA and two-thirds of these were formed ARA [46]. The above message suggests that the main type of oxylipins in various animals may be quite similar (specifically, ARA oxylipins). These oxylipins typically exhibit a greater variety and quantity, indicating their potential vital roles [45]. The oxylipins derived from N-6 polyunsaturated fatty acids (PUFAs) usually have proinflammatory, prothrombotic, and vasoconstrictive effects, but this is not absolute. On the contrary, the oxylipins derived from n-3 PUFAs often exhibit anti-inflammatory, vasodilatory, and antithrombotic effects [47,48]. Notably, in the 1.0% n-3 HUFA diet group, a substantial proportion of DHA-derived oxylipins, including 4-HDHA, 7-HDHA, 8-HDHA, 10-HDHA, 14(S)-HDHA, and 16-HDHA, reached their peak, indicating that DHA can be efficiently converted into biologically active oxylipins under these dietary conditions. Conversely, in the 2.0% n-3 HUFA diet group, although some oxylipins still peaked, the proportion was relatively lower, potentially due to the regulation of fatty acid metabolic pathways. Similarly, in the 2.0% n-3 HUFA diet group, over half of the EPA-derived oxylipins reached a peak, particularly 5-HEPE and 14(15)-EpETE, whose levels increased significantly. These oxylipins likely possess specific biological functions that positively impact the physiological state of Yangtze sturgeon. Moreover, in the 1% to 2% n-3 HUFA diet groups, the ARA-derived oxylipins also exhibited a marked increase, further corroborating the intimate link between oxylipin production and the composition of fatty acids. Similar research also suggests that the concurrent elevation of plasma EPA levels along with plasma EPA-oxylipin levels is consistent with previous intervention studies, indicating that the oxylipin pattern reflects changes in the parent PUFA [49]. These findings suggest that the peak responses of DHA- and EPA-derived oxylipins are primarily inconsistent, occurring within the range of 1–2% N-3 HUFAs. Furthermore, in diets containing 1–2% n-3 HUFAs, over 60% of ARA-derived oxylipin production also reached a peak. The peak response concentrations of these oxylipins mirror those observed in antioxidants and immune responses. The functions of these oxylipins significantly impact the antioxidant and immune status of the Yangtze sturgeon, with particular emphasis on the 14 oxylipins that exhibited significant increases, warranting further investigation. These oxylipins may maintain the Yangtze sturgeon’s health by modulating antioxidant and immune responses. It is noteworthy that our study identified 14 oxylipins with significantly elevated levels, which may exert unique effects on the antioxidant and immune status of the Yangtze sturgeon. Therefore, future studies could delve deeper into the specific mechanisms of action of these oxylipins and their interactions with other biological molecules in jointly regulating the physiological processes of the Yangtze sturgeon. Generally, our study offers profound insights into the effects of n-3 HUFAs on the production of oxylipins in Yangtze sturgeon, serving as a valuable reference for the breeding and nutritional management of this species. By fine-tuning feed formulations and nutritional strategies, we can effectively modulate the levels of oxylipins in the Yangtze sturgeon, thereby enhancing its antioxidant and immune statuses, improving breeding efficiency and ensuring biological safety.

## 5. Conclusions

Adding n-3 HUFAs to the diet of Yangtze sturgeon can significantly boost their antioxidant capacity by strengthening the muscular activities of T-AOC, SOD, and CAT while simultaneously reducing muscle MDA content. Moreover, dietary n-3 HUFAs effectively mitigate physiological stress by elevating serum biochemical indicators, such as ALP, LDLC, TG, TCHO, GSH-Px, and SOD. Notably, the peak levels of most oxylipins occur with 1% to 2% N-3 HUFAs, which aligns with the requirements for optimal antioxidant and immune responses. Consequently, we suggest incorporating n-3 HUFAs into the diet at a concentration of 1.0 to 2.0% to enhance both the antioxidant capacity and overall physiological status of the sturgeon. These insights provide invaluable nutritional guidance for safeguarding this endangered species during its crucial stage II gonadal development.

## Figures and Tables

**Table 1:** Formulation and ingredients of the basal diet (%).

Ingredients	Dietary Inclusion of n-3 Highly Unsaturated Fatty Acid Level (% of Diet)				
	0.50	1.00	1.50	2.00	2.40
Fish meal	36.00	36.00	36.00	36.00	36.00
Soybean meal	16.00	16.00	16.00	16.00	16.00
Chicken meal	10.00	10.00	10.00	10.00	10.00
Squid meal	5.00	5.00	5.00	5.00	5.00
ARA-enriched oil	0.50	0.50	0.50	0.50	0.50
Soybean oil	5.00	4.50	4.00	3.50	3.00
DHA-enriched oil	0.00	0.25	0.50	0.75	1.00
EPA-enriched oil	0.00	0.25	0.50	0.75	1.00
Wheat flour	21.20	21.20	21.20	21.20	21.20
Choline chloride	0.20	0.20	0.20	0.20	0.20
Monocalcium phosphate	1.00	1.00	1.00	1.00	1.0
Vitamin premix *	1.00	1.00	1.00	1.00	1.00
Mineral premix *., Guangdong, Shenzhen, China	2.00	2.00	2.00	2.00	2.00
Carboxymethylcellulose sodium	2.00	2.00	2.00	2.00	2.00
Mold inhibitor	0.05	0.05	0.05	0.05	0.05
Ethoxyquin	0.05	0.05	0.05	0.05	0.05
Proximate analysis (%)					
Moisture	6.60	7.41	7.99	7.62	6.90
Crude protein	42.39	42.33	42.79	43.33	41.87
Crude lipid	10.66	11.41	10.52	10.46	10.37
Ash	13.29	12.44	12.28	11.60	11.21

* The vitamin premixture provided the following per kg of diet: vitamin B_1_, 50 mg; vitamin B_2_, 200 mg; vitamin B_6_, 50 mg; vitamin B_12_, 20 mg; folic acid, 15 mg; vitamin C, 325 mg; calcium pantothenate, 400 mg; inositol, 1500 mg; D-biotin (2%), 5 mg; niacin, 750 mg; vitamin A, 2.5 mg; vitamin E, 160 mg; vitamin D3, 2.0 mg; and vitamin K_3_, 20 mg. * The mineral premixture provided the following per kg of diet: Ca (H_2_PO_4_)_2_, 1800 mg; KH_2_PO_4_, 1350 mg; NaCl, 500 mg; MgSO·7H_2_O, 750 mg; NaH_2_PO_4_·2H_2_O, 650 mg; KI, 1.5 mg; CoSo_4_·6H_2_O, 2.5 mg; CuSo_4_·5H_2_O, 15 mg; ZnSO_4_·7H_2_O, 350 mg; FeSO_4_·7H_2_O, 1250 mg; MnSO_4_·4H_2_O, 80 mg; and Na_2_SeO_3_, 6 mg.

**Table 2:** Fatty acid composition of the experimental diets with different levels of n-3 HUFAs (% total fatty acids).

Fatty Acid	Dietary n-3 HUFA Levels				
	0.50	1.00	1.50	2.00	2.40
C14: 0	1.08	1.05	0.88	1.26	1.08
C15: 0	0.15	0.15	0.13	0.14	0.14
C16: 0	16.72	15.53	14.61	14.52	14.09
C16: 1	2.03	2.13	2.03	2.38	2.08
C17: 0	0.14	0.15	0.15	0.00	0.15
C17: 1	0.14	0.00	0.00	0.00	0.00
C18: 0	4.55	4.26	4.18	4.05	3.80
C18: 1n9	21.47	20.00	18.99	17.19	16.80
C18: 2n6	37.59	35.67	33.46	29.21	26.12
C20: 0	0.25	0.23	0.23	0.23	0.21
C18: 3n6	0.00	0.02	0.11	0.13	0.11
C20: 1	1.65	1.46	1.75	0.92	1.26
C18: 3n3	3.84	3.62	3.46	3.06	2.76
C20: 2	0.13	0.15	0.14	0.17	0.14
C22: 0	0.31	0.27	0.31	0.27	0.25
C20: 3n-6	0.33	0.34	0.36	0.35	0.35
C20: 4n-6 (ARA)	2.74	2.90	2.84	3.02	2.86
C20: 5 (EPA)	2.16	5.47	8.02	12.62	15.44
C24: 0	0.40	0.35	0.50	0.63	0.60
C24: 1	0.19	0.17	0.24	0.21	0.19
C22: 6n-3 (DHA)	4.02	5.98	7.51	9.55	11.46
n-3 HUFA	6.18	11.45	15.53	22.17	26.90

ARA, arachidonic acid; DHA, docosahexaenoic acid; EPA, eicosapentaenoic acid; n-3 HUFAs, n-3 highly unsaturated fatty acids (EPA + DHA).

**Table 3:** Growth performance and feed utilization of female F_2_-generation Yangtze sturgeon fed diets containing different levels of n-3 HUFAs.

Fatty Acid	Dietary n-3 HUFA Levels					*p*-Value
	0.50	1.00	1.50	2.00	2.40	
IBW, (kg)	3.43 ± 0.29	3.81 ± 0.27	3.52 ± 0.34	3.77 ± 0.19	3.43 ± 0.07	0.717
IBL, (cm)	80.16 ± 3.66	88.83 ± 2.24	87.66 ± 2.74	89.83 ± 1.47	78.83 ± 2.89	0.057
FBW, (kg)	5.51 ± 0.34	6.26 ± 0.54	5.68 ± 0.39	6.12 ± 0.29	5.31 ± 0.29	0.411
FBL, (cm)	89.00 ± 1.77	90.83 ± 1.74	89.00 ± 1.93	93.50 ± 1.40	86.83 ± 1.07	0.079
WGR, (%)	61.99 ± 11.02	63.79 ± 2.52	62.01 ± 5.11	62.33 ± 1.20	54.58 ± 8.03	0.872
FCR	2.81 ± 0.40	2.38 ± 0.22	2.67 ± 0.07	2.41 ± 0.11	3.07 ± 0.43	0.457
CF, (%)	0.96 ± 0.08	0.90 ± 0.05	0.88 ± 0.10	0.97 ± 0.02	0.86 ± 0.06	0.576

Data for IBW, FBW, WGR, FCR, and survival rate are presented as the means ± SE (n = 15), and the data for IBL, FBL, and CF are also presented as the means ± SE (n = 6). IBW, initial body weight; IBL, initial body length; FBW, final body weight; FBL, final body length; WGR, weight gain rate; FCR, feed conversion ratio; CF, condition factor.

**Table 4:** Serum biochemical indexes of female F2-generation Yangtze sturgeon fed diets containing different levels of n-3 HUFAs.

Parameter	Dietary n-3 HUFA Levels					*p*-Value
	0.50	1.00	1.50	2.00	2.40	
AST, (U/L)	163.93 ± 13.90	161.33 ± 1.85	168.56 ± 12.67	152.90 ± 4.29	136.96 ± 9.27	0.232
ALT, (U/L)	21.83 ± 4.90	25.00 ± 4.27	28.56 ± 3.13	22.66 ± 5.46	13.80 ± 0.95	0.206
ALP, (U/L)	79.03 ± 2.00 ^a^	65.43 ± 6.75 ^ab^	67.53 ± 1.23 ^ab^	47.33 ± 1.08 ^c^	55.33 ± 3.75 ^bc^	0.001
ALB, (g/L)	10.70 ± 0.80	10.50 ± 0.20	11.73 ± 0.40	12.36 ± 0.73	11.63 ± 0.17	0.155
TP, (g/L)	32.02 ± 2.61	31.74 ± 1.06	33.00 ± 2.29	33.14 ± 1.42	32.41 ± 1.33	0.977
GLU, (mmol/L)	2.18 ± 0.26	2.24 ± 0.01	2.15 ± 0.18	2.19 ± 0.07	2.37 ± 0.10	0.863
HDLC, (mmol/L)	0.23 ± 0.03	0.19 ± 0.01	0.20 ± 0.00	0.21 ± 0.00	0.22 ± 0.02	0.624
LDLC, (mmol/L)	0.64 ± 0.05 ^a^	0.97 ± 0.01 ^b^	1.18 ± 0.12 ^b^	1.19 ± 0.00 ^b^	1.10 ± 0.02 ^b^	0.001
TG, (mmol/L)	2.03 ± 0.06 ^a^	2.42 ± 0.03 ^ab^	4.02 ± 0.07 ^c^	2.81 ± 0.16 ^b^	2.79 ± 0.04 ^b^	0.000
TCHO, (mmol/L)	1.37 ± 0.08 ^a^	1.86 ± 0.15 ^ab^	2.15 ± 0.17 ^b^	2.15 ± 0.17 ^b^	2.40 ± 0.12 ^b^	0.005

Data are presented as the means ± SE (n = 3). Data in the same row with different superscript letters are statistically significant (*p* < 0.05). AST, aspartate aminotransferase; ALT, alanine aminotransferase; ALP, alkaline phosphatase; ALB, albumin; TP, total protein; GLU, glucose; HDLC, high-density lipoprotein cholesterol; LDLC, low-density lipoprotein cholesterol; TCHO, total cholesterol; TGs, triglycerides.

**Table 5:** Serum antioxidant and immunity statuses of female F2-generation Yangtze sturgeon fed diets containing different levels of n-3 HUFAs.

Parameter	Dietary n-3 HUFA Levels					*p*-Value
	0.50	1.00	1.50	2.00	2.40	
Serum antioxidant						
T-AOC, (mM)	0.35 ± 0.00	0.41 ± 0.02	0.40 ± 0.00	0.41 ± 0.02	0.37 ± 0.00	0.071
SOD, (U/mL)	47.57 ± 2.95 ^a^	52.57 ± 1.94 ^ab^	51.92 ± 1.99 ^ab^	56.47 ± 1.94 ^b^	58.54 ± 1.02 ^b^	0.016
GSH-Px, (μmol/L)	281.76 ± 6.78 ^a^	307.05 ± 3.52 ^ab^	288.23 ± 25.04 ^a^	297.64 ± 19.07 ^ab^	362.35 ± 8.66 ^b^	0.023
CAT, (U/mL)	4.15 ± 0.63	3.58 ± 0.23	3.37 ± 0.21	4.51 ± 0.10	3.91 ± 0.10	0.177
MDA, (nmol/mL)	2.66 ± 0.09	2.38 ± 0.25	2.76 ± 0.09	3.52 ± 0.09	3.14 ± 0.43	0.128
Serum immunity						
IgM, (mg/L)	41.53 ± 3.37 ^a^	68.07 ± 4.32 ^b^	57.62 ± 3.06 ^ab^	59.17 ± 2.65 ^ab^	47.81 ± 8.48 ^ab^	0.024
C3, (mg/L)	41.29 ± 3.82	41.29 ± 3.82	39.41 ± 1.94	59.48 ± 6.02	48.70 ± 6.41	0.065
C4, (mg/L)	47.23 ± 0.73	46.48 ± 2.94	47.22 ± 2.65	50.91 ± 2.65	46.49 ± 1.47	0.628

Data are presented as the means ± SE (n = 3). Data in the same row with different superscript letters are statistically significant (*p* < 0.05). T-AOC, total antioxidant capacity; SOD, superoxide dismutase; GSH-Px, glutathione peroxidase; CAT, catalase; MDA: malondialdehyde; C3, complement 3; C4, complement 4; IgM, immunoglobulin M.

**Table 6:** Muscle antioxidant and immunity status of female F_2_-generation Yangtze sturgeon fed diets containing different levels of n-3 HUFAs.

Parameter	Dietary n-3 HUFA Levels					*p*-Value
	0.50	1.00	1.50	2.00	2.40	
Muscle antioxidant						
T-AOC, (mmol/g)	0.19 ± 0.03 ^a^	0.18 ± 0.001 ^a^	0.22 ± 0.00 ^a^	0.35 ± 0.02 ^b^	0.29 ± 0.03 ^ab^	0.006
SOD, (U/mgprot)	24.12 ± 1.68 ^a^	94.81 ± 2.37 ^c^	64.55 ± 11.03 ^bc^	67.62 ± 10.19 ^bc^	53.92 ± 7.73 ^ab^	0.001
CAT, (mgprot/mL)	2.60 ± 0.17 ^a^	8.24 ± 1.43 ^c^	7.11 ± 0.96 ^bc^	3.80 ± 0.07 ^abc^	3.40 ± 0.12 ^ab^	0.011
MDA, (nmol/mgprot)	4.73 ± 0.35 ^a^	5.00 ± 0.46 ^a^	5.42 ± 0.12 ^a^	2.68 ± 0.30 ^b^	2.58 ± 0.63 ^b^	0.001
Muscle immunity						
IgM, (mg/L)	69.58 ± 2.91	66.38 ± 7.70	62.23 ± 2.62	66.66 ± 2.91	52.97 ± 1.58	0.102
C3, (mg/L)	107.27 ± 2.54 ^ab^	109.82 ± 2.54 ^b^	112.25 ± 4.30 ^b^	99.38 ± 2.67 ^ab^	95.32 ± 1.39 ^a^	0.008
C4, (mg/L)	32.96 ± 2.03	27.50 ± 2.35	30.64 ± 1.55	27.49 ± 2.38	36.77 ± 2.26	0.054

Data are presented as the means ± SE (n = 3). Data in the same row with different superscript letters are statistically significant (*p* < 0.05). T-AOC, total antioxidant capacity; SOD, superoxide dismutase; CAT, catalase; MDA: malondialdehyde; C3, complement 3; C4, complement 4; IgM, immunoglobulin M.

**Table 7:** Serum oxylipin status of female F_2_-generation Yangtze sturgeon fed diets containing different levels of n-3 HUFAs (nmol/L).

Parameter	Dietary n-3 HUFA Levels					*p*-Value
	0.50	1.00	1.50	2.00	2.40	
PGA_2_	28.51 ± 5.62 ^b^	16.10 ± 2.53 ^ab^	13.01 ± 4.19 ^ab^	6.50 ± 1.00 ^a^	7.66 ± 3.57 ^a^	0.012
18-HETE	1.03 ± 0.25 ^a^	1.11 ± 0.41 ^ab^	1.21 ± 0.1 1 ^ab^	2.46 ± 0.43 ^b^	1.35 ± 0.05 ^ab^	0.037
17-HETE	1.06 ± 0.15 ^a^	1.10 ± 0.35 ^a^	2.53 ± 0.32 ^ab^	5.31 ± 1.09 ^b^	3.03 ± 0.80 ^ab^	0.005
D-γ-LA	21,994.5 ± 4879.64 ^ab^	30,731.86 ± 4205.54 ^b^	23,193 ± 1564.94 ^ab^	22,493.06 ± 3862.09 ^ab^	12,942.23 ± 1539.95 ^a^	0.059
16-HDHA	54.86 ± 9.70 ^a^	111.98 ± 3.24 ^b^	66.91 ± 6.94 ^ab^	68.27 ± 17.15 ^ab^	70.57 ± 4.94 ^ab^	0.017
10-HDHA	49.63 ± 8.41 ^a^	105.05 ± 8.46 ^b^	66.46 ± 6.25 ^a^	62.76 ± 9.5 ^a^	55.61 ± 4.75 ^a^	0.004
14(S)-HDHA	60.1 ± 11.88 ^a^	117.42 ± 12.49 ^b^	63.63 ± 6.18 ^a^	64.44 ± 15.32 ^a^	63.68 ± 4.20 ^a^	0.017
7-HDHA	32.28 ± 3.71 ^a^	97.53 ± 4.89 ^c^	65.00 ± 5.19 ^b^	60.30 ± 10.14 ^ab^	48.43 ± 6.28 ^ab^	0.000
8-HDHA	63.47 ± 9.43 ^a^	283.26 ± 15.88 ^c^	178.34 ± 16.58 ^b^	139.82 ± 32.13 ^ab^	98.79 ± 29.01 ^ab^	0.000
4-HDHA	125.93 ± 13.93 ^a^	338.92 ± 18.96 ^c^	230.30 ± 30.12 ^b^	187.72 ± 20.41 ^ab^	127.55 ± 6.62 ^a^	0.000
5-HEPE	27.57 ± 2.69 ^a^	39.93 ± 7.18 ^ab^	47.55 ± 3.07 ^ab^	53.70 ± 5.49 ^b^	45.90 ± 4.23 ^ab^	0.030
14(15)-EpETE	785.27 ± 289.52 ^a^	721.89 ± 441.70 ^a^	953.26 ± 325.63 ^ab^	3477.85 ± 773.97 ^b^	1621.54 ± 815.48 ^ab^	0.033
EPA	18,257.73 ± 2409.31 ^a^	32,906.33 ± 3228.87 ^ab^	32,951.86 ± 1873.49 ^ab^	46,873.29 ± 12,076.60 ^ab^	60,478.80 ± 20,694.81 ^b^	0.030
γ-LA	7429.47 ± 1602.35 ^ab^	9924.74 ± 1805.85 ^b^	6559.60 ± 341.76 ^ab^	5871.33 ± 845.72 ^ab^	2545.32 ± 23.47 ^a^	0.014

Data are presented as the means ± SE (n = 3). Data in the same row with different superscript letters are statistically significant (*p* < 0.05).

## Data Availability

Data in present study are contained within the manuscript and Supplementary Materials.

## Supplementary Materials

The following supporting information can be downloaded at: https://www.mdpi.com/article/10.3390/antiox13040421/s1. Figure S1: The Yangtze sturgeon histological section of the stage II ovary; Figure S2: The amplified band of Yangtze sturgeon; Figure S3: The quality control of LC-MS/MS used in this study. (A) Total ions current (TIC) overlap plot of quality control samples; (B) Integral correction diagram; (C) Canonical plotting; Table S1: Liner equation and the profile of oxylipins in Yangtze sturgeon fed n-3 highly unsaturated fatty acids diets; Table S2: Classification of 62 oxylipins in Yangtze sturgeon fed n-3 highly unsaturated fatty acids diets.
