# Supplementary material for: The Effects of Dietary n-3 Highly Unsaturated Fatty Acids on Growth, Antioxidant Capacity, Immunity, and Oxylipin Profiles in Acipenser dabryanus
Source: Antioxidants (Basel). 2024 Mar 29;13(4):421. doi: 10.3390/antiox13040421 (PMC11047622; doi:10.3390/antiox13040421)
Supplement: Supplementary file 1 [file antioxidants-13-00421-s001.zip › Supplementary Figure.pdf]

**Figure S1** The Yangtze sturgeon histological section of the stage II ovary.

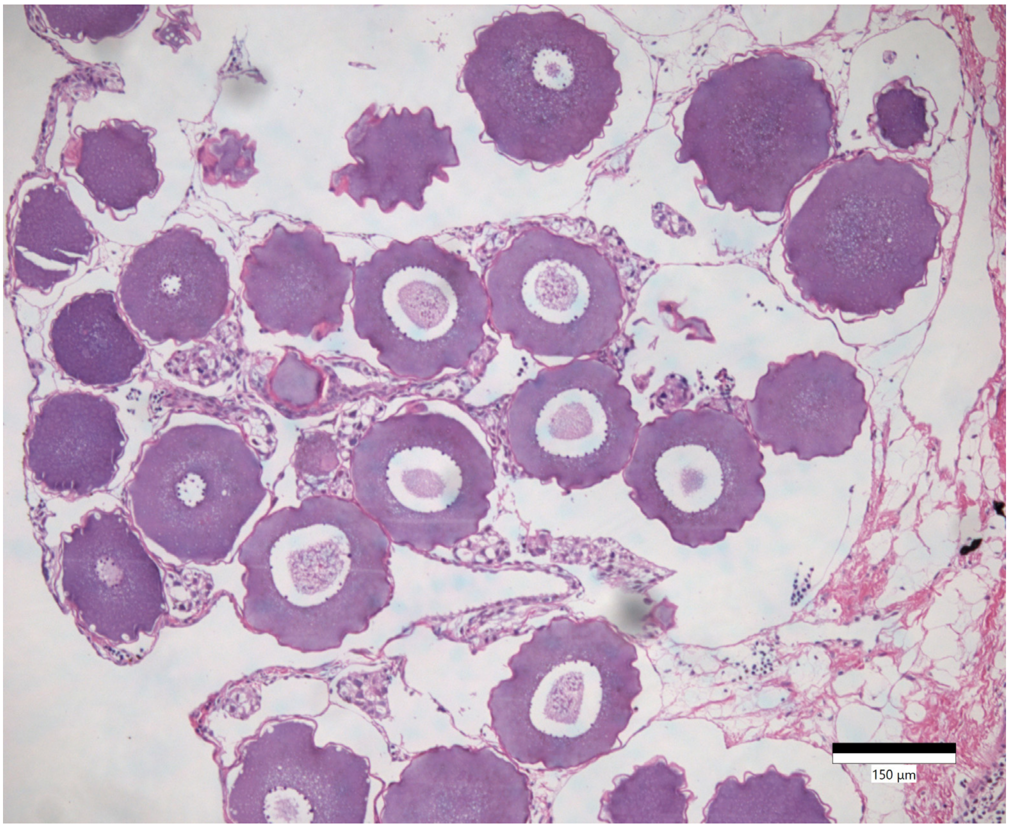


**Figure S2** The amplified band of Yangtze sturgeon.

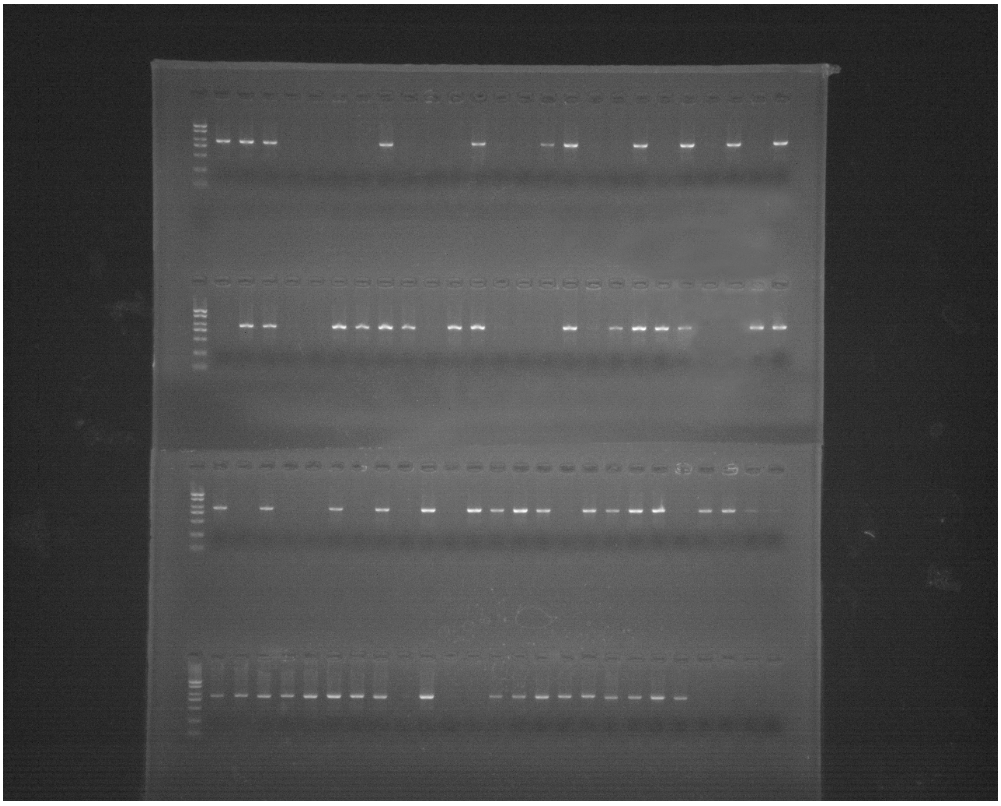

**Figure S3** The quality control of LC-MS/MS used in this study. **(A)** Total ions current (TIC) overlap plot of quality control samples; **(B)** Integral correction diagram; **(C)** Canonical plotting.

**(A)**

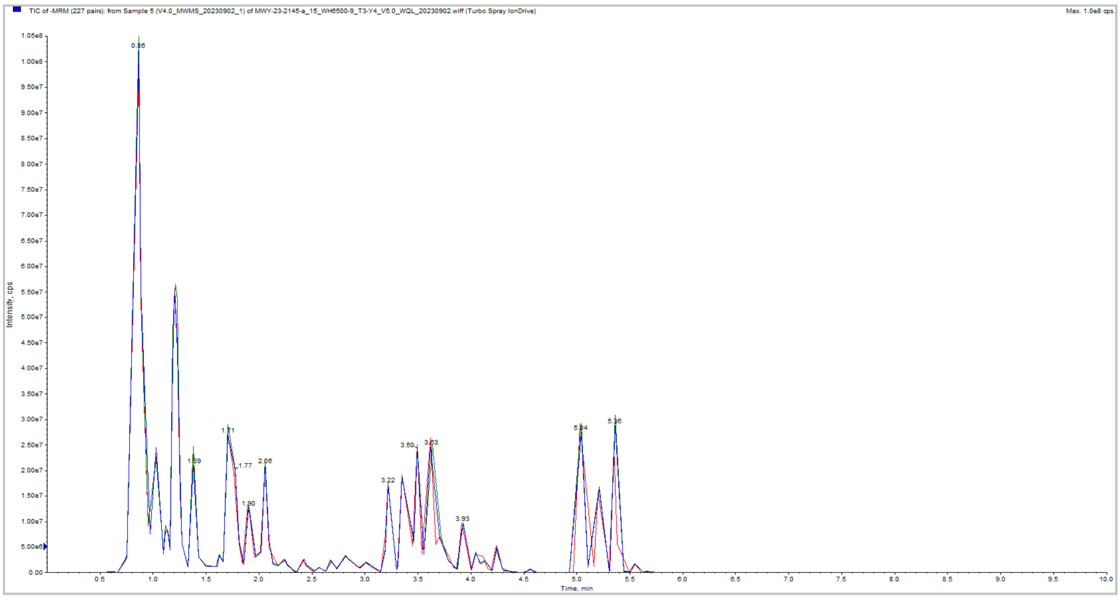


**(B)**

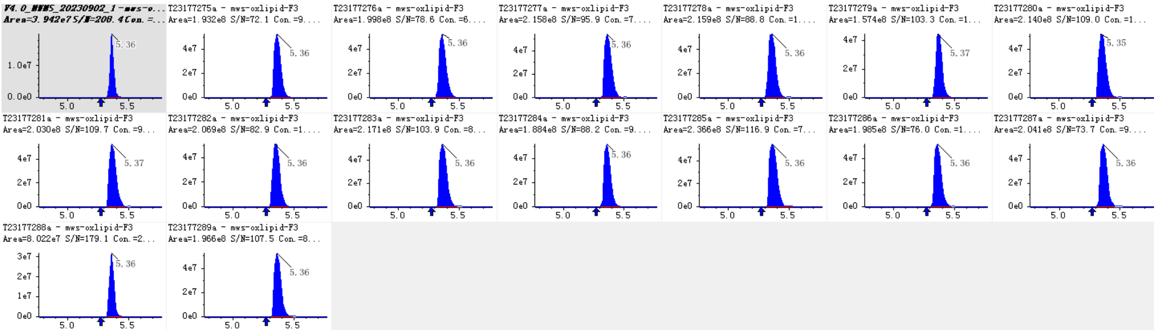

(C)

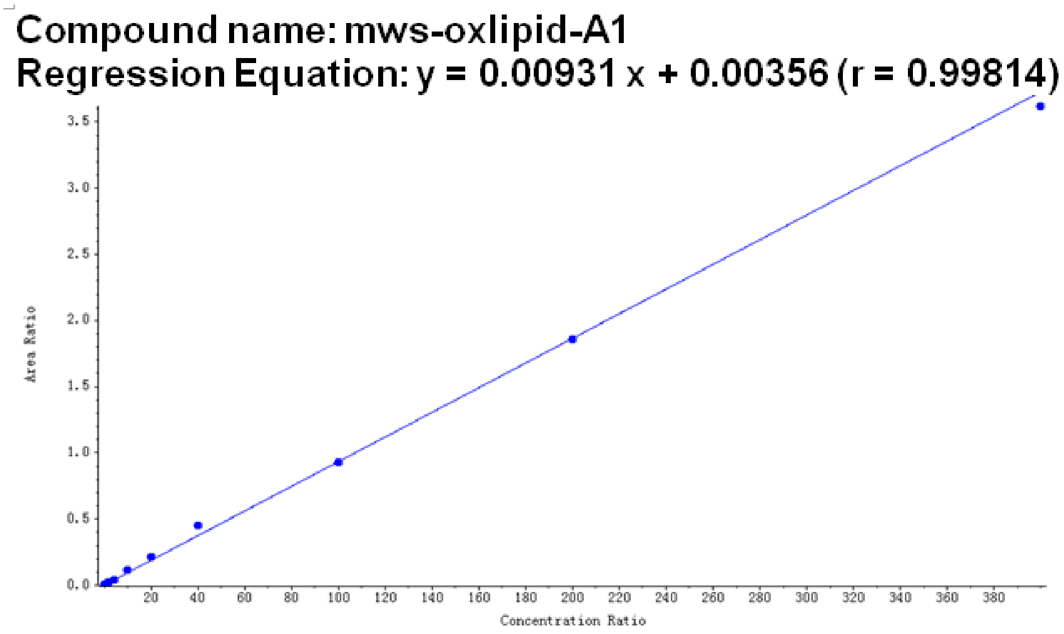
Compound name: mws-oxlipid-A1
Regression Equation: y = 0.00931 x + 0.00356 (r = 0.99814)
Area Ratio
3.5
3.0
2.5
2.0
1.5
1.0
0.5
0.0
20 40 60 80 100 120 140 160 180 200 220 240 260 280 300 320 340 360 380
Concentration Ratio

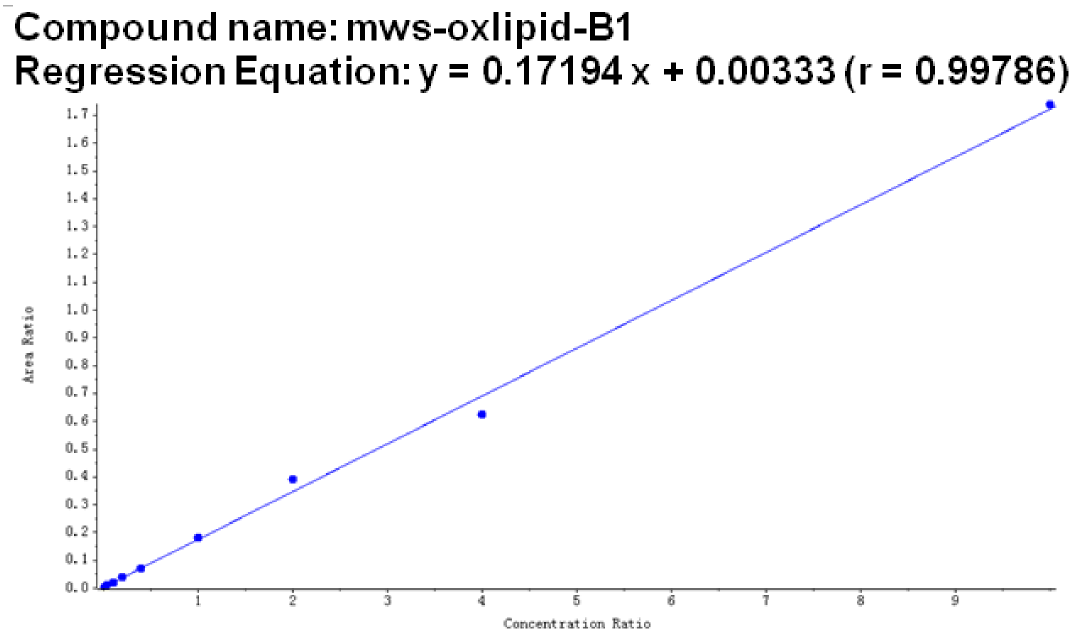
Compound name: mws-oxlipid-B1
Regression Equation: y = 0.17194 x + 0.00333 (r = 0.99786)
Area Ratio
1.7
1.6
1.5
1.4
1.3
1.2
1.1
1.0
0.9
0.8
0.7
0.6
0.5
0.4
0.3
0.2
0.1
0.0
1 2 3 4 5 6 7 8 9
Concentration Ratio
