# Supplementary material for: The Effects of Dietary n-3 Highly Unsaturated Fatty Acids on Growth, Antioxidant Capacity, Immunity, and Oxylipin Profiles in Acipenser dabryanus
Source: Antioxidants (Basel). 2024 Mar 29;13(4):421. doi: 10.3390/antiox13040421 (PMC11047622; doi:10.3390/antiox13040421)
Supplement: Supplementary file 1 [file antioxidants-13-00421-s001.zip › Supplementary Table.pdf]

Table S1. Liner equation and the profile of oxylipins in Yangtze sturgeon fed n-3 highly unsaturated fatty acids diets (nM/L; n=3, mean vaule)

Index	Class	Equation	r	Dietary inclusion of n-3 highly unsaturated fatty acid level (% of diet)					LLOQ	ULOQ
				0.50%	1.00%	1.50%	2.00%	2.40%		
11-HEDE	-	y = 0.01178 x - 3.11630e-4	0.99800	12.6	4.97	4.66	7.98	6.39	0.2	400
15-HEDE	-	y = 0.01381 x - 0.00128	0.99393	103.20	14.52	8.91	38.25	50.80	0.2	400
13-HOTrE	ALA	y = 0.00719 x - 2.57666e-4	0.99549	254.98	20.94	18.39	174.77	273.24	0.2	400
9(S)-HpOTrE	ALA	y = 0.00999 x - 0.00168	0.99558	47.21	25.23	26.46	43.80	18.51	0.2	400
9-OxoOTrE	ALA	y = 0.01012 x + 0.00341	0.99357	2.13	1.24	1.76	1.83	1.09	0.2	400
α-LA	ALA	y = 0.12160 x + 0.01238	0.99821	9505.94	10059.31	10259.05	9321.25	5866.88	40	40000
20-hydroxyPGF2α	ARA	y = 0.00998 x + 6.42569e-4	0.99960	1.92	4.62	1.83	1.74	4.64	1	400
6keto-PGF1α	ARA	y = 0.01206 x + 6.38911e-4	0.99705	5.11	5.83	5.35	4.78	7.02	0.2	400
5-isoPGF2VI	ARA	y = 0.62607 x + 0.01282	0.99790	0.77	0.42	0.36	0.09	0.61	0.2	400
11β-PGF2α	ARA	y = 0.51412 x + 0.01338	0.99916	0.75	0.39	0.34	0.08	0.59	1	400
8-iso-PGF2α	ARA	y = 0.46647 x + 0.00998	0.99791	85.72	44.48	37.58	12.70	67.26	10	400
PGE2	ARA	y = 0.02384 x + 0.00123	0.99144	9.15	5.37	4.54	3.21	6.19	0.2	400
11β-PGE2	ARA	y = 0.02953 x + 9.09588e-4	0.99867	7.15	4.49	3.82	2.67	3.83	0.4	400
15-keto-PGF2α	ARA	y = 0.04604 x + 0.00166	0.99920	1.87	1.31	1.03	0.73	1.18	4	400
PGA2	ARA	y = 0.00636 x + 0.00117	0.99745	28.51	16.10	13.01	6.50	7.66	0.4	400
tetranor-12(S)-HETE	ARA	y = 0.01895 x + 3.40177e-4	0.99940	0.14	0.20	0.12	0.18	0.11	0.2	400
14(15)-DiHET	ARA	y = 0.09944 x + 0.00688	0.99959	19.31	18.18	13.55	26.24	8.32	0.2	400
12-HHT	ARA	y = 0.00272 x - 1.70150e-4	0.99553	0.77	2.40	0.59	0.65	0.79	0.4	400

11(12)-DiHET	ARA	y = 0.04910 x + 0.00350	0.99834	12.98	12.49	9.03	17.93	5.80	0.2	400
20-COOH-AA	ARA	y = 0.06452 x - 0.00104	0.99273	9.96	9.86	10.78	11.20	7.01	0.4	400
8(9)-DiHETE	ARA	y = 0.01823 x - 5.32128e-5	0.99968	1.32	1.41	2.35	6.97	3.09	0.2	400
5,6-DiHETrE	ARA	y = 0.11782 x + 0.00554	0.99375	3.48	3.30	2.51	4.21	1.56	0.2	400
20-HETE	ARA	y = 0.01080 x + 2.27241e-4	0.99938	0.89	0.82	0.97	1.13	0.70	0.2	400
18-HETE	ARA	y = 0.01829 x + 3.09395e-4	0.99917	1.03	1.11	1.21	2.46	1.35	0.2	400
17-HETE	ARA	y = 0.01811 x + 2.63373e-4	0.99923	1.06	1.10	2.53	5.31	3.03	0.2	400
16-HETE	ARA	y = 0.00773 x + 1.62531e-4	0.99932	3.24	3.03	2.81	3.49	2.00	0.2	400
15-HETE	ARA	y = 0.16819 x + 0.00636	0.99599	309.73	97.96	41.24	189.80	159.01	2	4000
11(S)-HETE	ARA	y = 0.42145 x + 0.01434	0.99637	21.32	21.37	12.15	17.14	12.37	2	4000
8-HETE	ARA	y = 0.16318 x + 0.00493	0.99537	16.62	20.31	10.65	14.28	9.22	2	4000
12-HETE	ARA	y = 0.15414 x + 0.00362	0.99690	37.54	34.61	17.79	23.59	15.52	2	4000
5-HETE	ARA	y = 0.02700 x + 1.09194e-4	0.99854	29.71	38.63	33.66	31.68	18.30	4	4000
14,15-EET	ARA	y = 0.00931 x + 0.00356	0.99814	2500.65	1769.35	1252.24	3139.09	1029.91	0.2	400
5-oxoETE	ARA	y = 0.13437 x + 0.00779	0.99916	19.57	21.60	14.39	21.26	7.99	0.2	400
11,12-EET	ARA	y = 0.00350 x + 0.00150	0.99893	1575.64	1216.18	961.99	1965.22	687.03	0.2	400
8,9-EET	ARA	y = 0.00519 x + 0.00107	0.99933	579.1	437.10	328.99	702.19	235.10	0.2	400
5,6-EET	ARA	y = 2.57205e-4 x + 5.26942e-5	0.99944	14806.26	11231.76	8792.54	19224.09	6562.05	1	400
AA	ARA	y = 0.03653 x + 0.00332	0.99901	54617.06	86944.10	56630.00	66650.01	52481.40	40	40000
11-deoxyPGE1	DGLA	y = 0.00525 x + 0.00182	0.99962	159.47	219.35	118.44	236.63	101.37	0.2	400
15(S)-HETrE	DGLA	y = 0.04118 x + 0.00231	0.99874	103	20.36	10.29	40.28	37.55	0.2	400
8(S)-HETrE	DGLA	y = 0.02343 x - 7.07952e-4	0.99930	3.39	4.25	2.98	2.61	2.40	0.2	400
D-γ-LA	DGLA	y = 0.02922 x + 0.00131	0.99150	21994.5	30731.86	23193	22493.06	12942.23	40	40000

19(20)-DiHDPE(A)	DHA	y = 0.00807 x + 4.98010e-4	0.99904	38.33	33.68	34.35	72.19	31.99	4	400
13(14)-DiHDPE(A)	DHA	y = 0.06455 x + 0.00182	0.99952	9.45	7.65	7.59	17.72	7.42	0.2	400
7(8)-DiHDPE(A)	DHA	y = 0.02011 x + 5.93627e-4	0.99954	7.88	6.41	6.41	14.5	6.38	0.2	400
20-HDHA	DHA	y = 0.04026 x + 2.10443e-4	0.99528	116.15	197.25	117.29	124.94	134.53	2	4000
16-HDHA	DHA	y = 0.13276 x - 0.00232	0.99913	54.86	111.98	66.91	68.27	70.57	1	4000
17-HDHA	DHA	y = 0.01744 x - 0.00115	0.99870	8813.53	1544.97	809.88	5493.69	7695.47	4	4000
13-HDHA	DHA	y = 0.04247 x - 0.00330	0.99899	273.23	105.50	55.07	211.70	245.93	1	4000
10-HDHA	DHA	y = 0.10069 x + 5.49150e-4	0.99900	49.63	105.05	66.46	62.76	55.61	1	4000
14(S)-HDHA	DHA	y = 0.04401 x + 7.91509e-5	0.99802	60.1	117.42	63.63	64.44	63.68	4	4000
7-HDHA	DHA	y = 0.05313 x + 1.34606e-4	0.99747	32.28	97.53	65.00	60.30	48.43	4	4000
8-HDHA	DHA	y = 0.03160 x - 0.00248	0.99860	63.47	283.26	178.34	139.82	98.79	1	4000
19(20)-EpDPE(A)	DHA	y = 0.11043 x + 0.01962	0.99214	1520.91	1106.22	934.55	2634.10	1209.07	0.2	400
4-HDHA	DHA	y = 0.05120 x + 5.55228e-5	0.99561	125.93	338.92	230.30	187.72	127.55	4	4000
16(17)-EpDPE	DHA	y = 0.04561 x + 0.01065	0.99895	3567.45	2259.00	2063.21	6022.34	2792.33	0.2	400
7,8-EpDPE	DHA	y = 0.07175 x + 0.02128	0.99555	4095.09	2774.55	2518.40	6960.96	3181.13	0.2	400
DHA	DHA	y = 0.18075 x + 0.02004	0.99722	77575.4	141307.50	99673.56	116429.97	131289.60	40	40000
RvE1	EPA	y = 0.01982 x + 0.00306	0.99367	6.46	45.82	4.70	8.90	95.38	0.2	400
PGF3α	EPA	y = 0.00890 x + 0.00139	0.99662	121.19	234.95	113.90	138.44	109.85	10	400
17(18)-DiHETE	EPA	y = 0.04310 x + 0.00103	0.99676	5.01	5.37	9.38	24.74	11.76	0.2	400
14(15)-DiHETE	EPA	y = 0.10046 x + 0.00180	0.99713	1.41	1.54	2.41	7.13	3.12	0.2	400
5,6-DIHETE	EPA	y = 0.02281 x + 5.85342e-4	0.99824	40.05	47.28	74.86	167.84	93.71	1	400
15-HEPE	EPA	y = 0.01335 x + 3.55801e-4	0.99412	355.53	64.81	51.43	970.72	1175.33	0.2	400
11-HEPE	EPA	y = 0.00625 x + 2.38816e-4	0.99539	415.54	72.30	60.14	1090.34	1344.58	1	400

12-HEPE	EPA	y = 0.01283 x + 2.23645e-4	0.99958	11.29	11.23	11.44	17.34	15.41	0.2	400
5-HEPE	EPA	y = 0.01093 x + 2.45261e-4	0.99683	27.57	39.93	47.55	53.70	45.90	0.2	400
17(18)-EpETE	EPA	y = 0.00755 x - 7.12118e-4	0.99925	42.3	60.54	81.09	125.26	72.39	0.2	400
14(15)-EpETE	EPA	y = 0.07717 x + 0.00362	0.99854	785.27	721.89	953.26	3477.85	1621.54	0.2	400
EPA	EPA	y = 0.35775 x + 0.01154	0.99782	18257.73	32906.33	32951.86	46873.29	60478.80	40	40000
γ-LA	GLA	y = 1.23316 x + 0.03901	0.99687	7429.47	9924.74	6559.60	5871.33	2545.32	40	40000
9(S),10(S),13(S)-TriHOME	LA	y = 0.04598 x + 0.00277	0.99721	5.16	2.52	4.88	3.04	4.94	0.2	400
12(13)-DiHOME	LA	y = 0.00822 x + 7.59501e-5	0.99873	24.5	16.08	15.60	31.03	10.03	0.2	400
9,10-DiHOME	LA	y = 0.01195 x + 7.33715e-4	0.99809	26.52	17.26	16.64	31.97	10.55	0.2	400
trans-EKODE-(E)-Ib	LA	y = 0.03332 x + 0.00106	0.99809	54.41	32.12	32.22	51.82	22.99	0.2	400
13(S)-HODE	LA	y = 0.05979 x + 0.00289	0.99966	197.31	44.58	32.66	102.74	116.09	0.2	400
9-HODE	LA	y = 0.07663 x + 0.00834	0.99636	125.1	35.74	30.11	76.50	77.83	0.2	400
12,13-EpOME	LA	y = 0.16569 x + 0.02561	0.99647	1461.9	949.73	846.91	1779.13	674.16	0.2	400
9,10-EpOME	LA	y = 0.21079 x + 0.01557	0.99428	1295.01	819.43	724.68	1508.83	582.99	0.2	400
LA	LA	y = 0.10290 x + 0.00794	0.99939	20060.5	28861.83	22015.30	23693.63	18802.76	40	40000
5-HETrE	MA	y = 0.02356 x - 4.63573e-4	0.99832	0.37	0.62	0.46	0.99	0.31	0.2	400

Table S2. Classification of 62 oxylipins in Yangtze sturgeon fed n-3 highly unsaturated fatty acids diets

Index	Sub class
8-iso-PGF2α	1
PGA2	1
5-isoPGF2VI	1
PGE2	1
11β-PGF2α	1
11β-PGE2	1
15-keto-PGF2α	1
9(S),10(S),13(S)-TriHOME	1
9(S)-HpOTrE	2
8,9-EET	2
5,6-EET	2
5,6-DiHETrE	2
14,15-EET	2
14(15)-DiHET	2
11,12-EET	2
11(12)-DiHET	2
12(13)-DiHOME	2
12,13-EpOME	2
trans-EKODE-(E)-Ib	2
9,10-EpOME	2
9,10-DiHOME	2
7(8)-DiHDPE(A)	3
7,8-EpDPE	3
13(14)-DiHDPE(A)	3
19(20)-EpDPE(A)	3
16(17)-EpDPE	3
19(20)-DiHDPE(A)	3
17-HETE	4
5,6-DIHETE	4
17(18)-EpETE	4
18-HETE	5
17(18)-DiHETE	5
14(15)-EpETE	5
8(9)-DiHETE	5
14(15)-DiHETE	5

11-deoxyPGE1	6
5-HETrE	6
12-HHT	7
10-HDHA	7
4-HDHA	7
7-HDHA	7
8-HDHA	7
16-HDHA	7
PGF3α	7
15-HEPE	8
11-HEPE	8
EPA	8
RvE1	8
15-HEDE	9
11-HEDE	9
13-HOTrE	9
15-HETE	9
15(S)-HETrE	9
17-HDHA	9
13(S)-HODE	9
9-HODE	9
8-HETE	10
5-oxoETE	10
5-HETE	10
12-HETE	10
D-γ-LA	10
γ-LA	10
